# Retraction: The prognostic value of prognostic nutritional index in hepatocellular carcinoma patients: A meta-analysis of observational studies

**DOI:** 10.1371/journal.pone.0273618

**Published:** 2022-08-22

**Authors:** The PLOS ONE Editors

After this article was published, similarities were noted between this article and submissions by other research groups, which call into question the validity and provenance of the reported results. In light of these issues, the *PLOS ONE* Editors retract this article [1].

PW did not agree with retraction. ZW and JW either did not respond or could not be reached directly to comment on the retraction decision.
